# A social ecological approach to promote learning health disparities in the clinical years: impact of a home-visiting educational program for medical students

**DOI:** 10.1186/s12909-022-03755-3

**Published:** 2022-09-30

**Authors:** Doron Sagi, Mary Catharine Joy Rudolf, Sivan Spitzer

**Affiliations:** 1 Azrieli Faculty of Medicine, Bar-Ilan University, 8 Henrietta Szold, 1311502 Safed, Israel; 2 MSR- The Israel Center for Medical Simulation, Sheba Medical Center, Tel-Hashomer, Israel

**Keywords:** Experiential learning, Social determinants of health, Healthcare disparities, Social ecology, Home visit, Pre-graduate medical education

## Abstract

**Background:**

There is consensus that medical schools have a duty to educate students about social determinants of health (SDOH) and equip them with skills required to ameliorate health disparities. Although the National Academy of Medicine (NAM) urged the development of experiential long term programs, teaching is usually conducted in the pre-clinical years or as voluntary courses. ETGAR a required health disparities course, based on the social ecological model, was initiated to answer the NAM call. This study aimed to ascertain the course impact on students learning of SDOH and health disparities.

**Methods:**

Students during their first clinical year cared for four patients in their transition from hospital back home, one patient in each internal medicine, surgery, pediatrics and obstetrics/gynecology rotation. The students home-visited their patients after meeting them in hospital and preparing a plain language discharge letter. Training session prior to the course, a tutorial in each rotation, and structured feedback gave the educational envelope. Mixed methodology was employed to evaluate the course impact. Quantitative data collected by students during the home-visit: patients’ characteristics and quality and safety of the transition back home using the Medication Discrepancy Tool and Care Transition Measure questionnaire. Stakeholders’ views were collected via interviews and focus groups with students representing all affiliated hospitals, and interviews with heads of departments most involved in the course.

**Results:**

Three hundred six students in three academic years, between October 2016–July 2019, completed home visits for 485 disadvantaged patients with improvement in patients’ knowledge of their treatment (3.2 (0.96) vs 3.8 (0.57), Z = -7.12, *p* < .0001) and identification of medication discrepancies in 42% of visits. Four themes emerged from the qualitative analysis: contribution to learning, experience-based learning, professional identity formation, and course implementation.

**Conclusions:**

ETGAR was perceived to complement hospital-based learning, making students witness the interaction between patients’ circumstances and health and exposing them to four patients’ environment levels. It provided a didactic framework for promoting awareness to SDOH and tools and behaviors required to ameliorate their impact on health and health disparities.

The course combined communication and community learning into traditionally bio-medical clinical years and serves as a model for how social-ecology approaches can be integrated into the curriculum.

**Supplementary Information:**

The online version contains supplementary material available at 10.1186/s12909-022-03755-3.

## Background

Health disparities are a source of public concern, and although most of the causes are external to health systems [1], the role and responsibilities of physicians are evident [2–4]. Health professionals have a responsibility to work to reduce disparities [5] and the range of skills required of physicians needs to be expanded. A report by the National Academy of Medicine (NAM) urges medical schools to develop a coherent, consistent and holistic framework, in partnership with the community, to educate and train physicians in this area [6] and equip students with the necessary skills to identify social determinants of health (SDOH) and address them [7].

Most medical schools teach communication and cross-cultural skills as a strategy to address the issue of disparities and SDOH during the pre-clinical years [6, 8, 9] As per the American College of Physicians, providing medical students with knowledge regarding SDOH and specific skills to tackle them is essential but insufficient [10] According to the NAM, education in this area needs to be experiential and integrated across the learning continuum [6] Most experience-based program involves voluntary courses for selected group of students such as University of New-Mexico HERO and PRIME-US in the University of California [3], or voluntary service-learning courses such as student-run clinics [11]. One longitudinal required course we identified, used reflective writing and simulation only as educational tools, without involving ‘real’ patients in a community setting [12]. The educational program we identified lacked the combination of being an experiential long term required course involving real patient learning.

Clinical rotations, the critical period where lifelong habits are developed, are likely to be a good setting for reinforcing didactic experiences and integrating skills into practice [8]. However, without intentional teaching, encounters with disadvantaged patients may not promote understanding or empathy for the difficulties they face and may even lead to the eroding of positive attitudes towards vulnerable populations [13, 14].

This study intends to ascertain the impact of an innovative educational program aimed to adhere the NAM guidelines for health disparity educational programs. ETGAR, an experienced-based course for clinical years students, was introduced in 2015 at Bar-Ilan University’s Faculty of Medicine and is now a required course. ETGAR- the Hebrew word for challenge is an acronym for health literacy, support and a bridge between medicine and community. ETGAR as service-learning aims to further students’ understanding of SDOH and health inequities and provide them with skills through working with patients in their transition from hospital back home [15]. Visiting patients’ homes, exposes students to social and personal determinants that are often not evident in hospital, and are crucial to fully understanding patients’ circumstances [16]. Home visits following discharge, a time when patients are most vulnerable to problems associated with SDOH [17], makes this experience potentially richer.

**Fig. 1:**
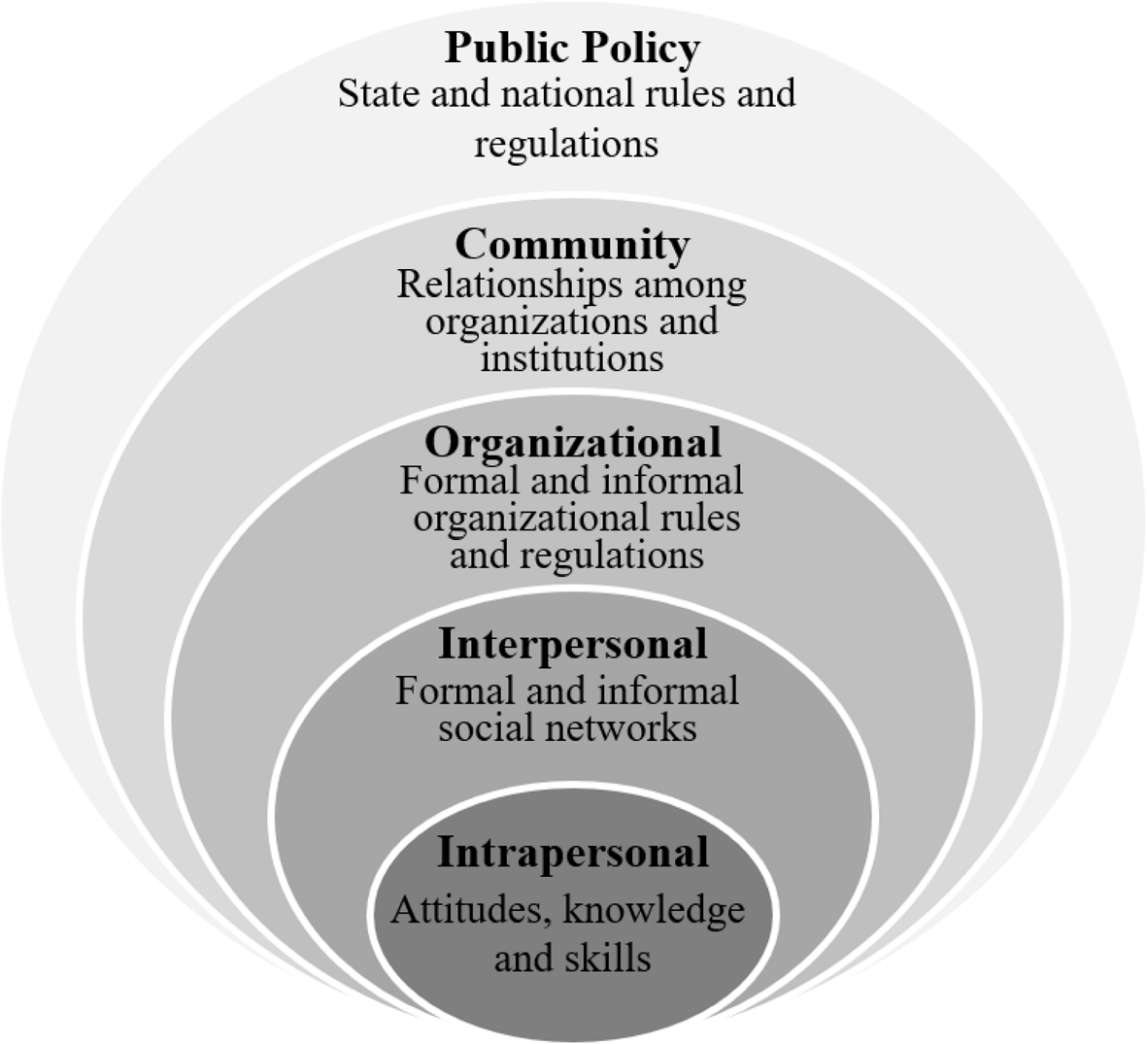
schematic description of the social ecological model of health. Adapted from Mcleroy et al. 1988 [21].

The course was developed utilizing the social-ecology approach for health promotion, which considers environmental influences on health and health behaviors [18], and the complex interrelated factors associated with inequities [19, 20]. We adopted McLeroy et al’s model which involves five levels: intrapersonal, interpersonal, institutional/organizational, community, and public policy [21] (Fig. 1), and used it to direct the development of ETGAR by taking students out of hospital in their first clinical year to conduct home visits with patients they encountered in hospital. Through preparing and conducting home visits, we anticipated students would go beyond their focus on biomedical factors and would gain an understanding of SDOH on at least three environment levels: intrapersonal, interpersonal, and organizational levels. Home visits also aimed to expose students to the interplay between hospital and community care and to practice the patient-centered skills required to address SDOH and health inequities.

We have already reported our investigation on two major educational tools developed and implemented in ETGAR: the post discharge home visit [22] and plain-language discharge letter [23]. The present study aimed a- to demonstrate the use of the social ecological model for developing a social disparity educational course. It also aimed to complete the picture by ascertaining ETGAR’s broader impact on students’ learning by b- assessing the course impact on students understanding of SDOH, health disparities and patients’ condition in the community; c- Ascertaining whether students adopted communication skills required to tackle health disparities.

## Methods

### Setting and participants

Bar-Ilan University’s Faculty of Medicine is in the Galilee in Israel’s northern periphery. Its affiliated hospitals serve a population of 1.4 million ethnically diverse and low-socioeconomic residents, among whom health inequities in morbidity and mortality as well as access and quality of care are prevalent compared with other regions of Israel [24]. Our study involved the 306 clinical students who participated in the required service learning between October 2016–July 2019, and were on rotation in four affiliated hospitals.

### The ETGAR course

**Fig. 2:**
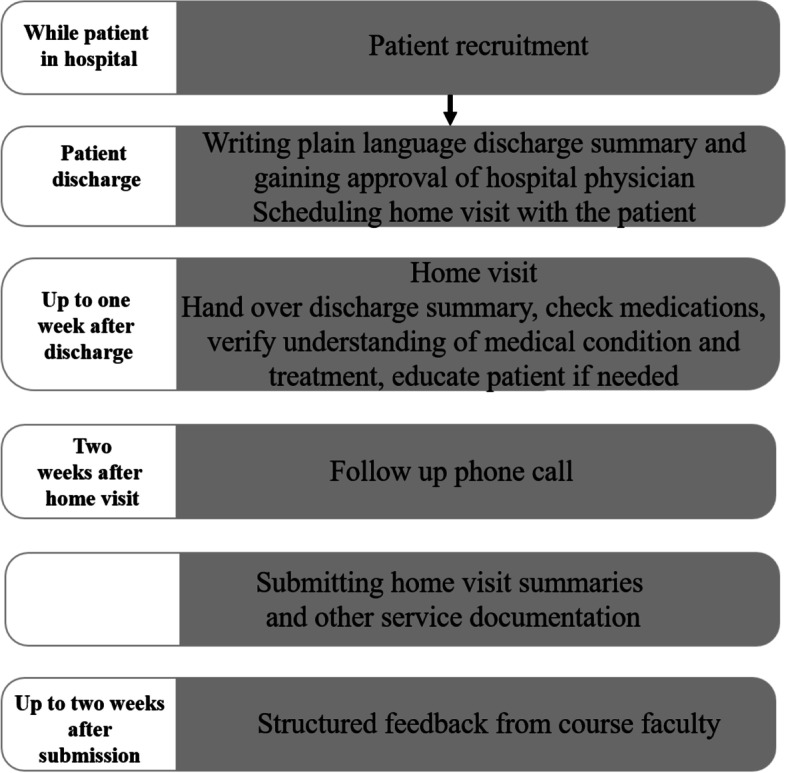
Schematic description of the ETGAR course

Students in their first clinical year are required to support four patients during their transition from hospital back home in: internal medicine, surgery, obstetrics/gynecology, and pediatrics, as previously described [25]. The patients are identified by staff or students as needing support for medical, social and/or personal circumstances. Figure 2 provides a schematic description of the service. Students chooses a peer from their rotation group to work in pairs or threes, in case of an odd number of students in the group. On completion of each visit each pair submit a report and their plain-language discharge letter and receive structured feedback.

Students could take an additional track of ETGAR caring for four or eight patients, in addition to the required course patients, for a scholarship. Since this scholarship track was external to the required course, patient consent was required for the student service and to be contacted by an independent researcher. One hundred twenty-nine students chose this track. The authors developed the course and conducted the pre-course training and the tutorials. SS and MCJR supervised the course.

Developing the course, we aimed to follow the socio-ecological approach principles by exposing students to the various environment levels: *intrapersonal* level (literacy and medical condition) when writing plain-language discharge letters. Home visits exposed students to the *interpersonal* level: support system, living conditions and socioeconomic status, and to the *organizational* level: community-based care. Meeting patients both in hospital and at home added exposure to the *community* level and the interplay between hospital and community care.

Students undergo a full-day training at the start of the course [25], and have four tutorials during their clinical rotations, aimed at exploring their experiences, and reinforcing: plain-language writing skills, checking medications, and knowledge of community services. To stand the course duties students are expected to attend the training and tutorial sessions, and to serve four patients including the submission of full home visit reports.

For scholarship students’ patients only, additional follow up phone interview was done four to 6 weeks after the home visit. A structured interview conducted by an independent interviewer containing open questions regarding the visit’s contribution to patients’ understanding and management of their medical condition, and a short questionnaire where patients graded their experience of home visit and transition in care.

### Research methods

This research is a form of action research [26], as the authors consists of the ETGAR team. The research was undertaken as a step towards extending the understanding of ETGAR as an educational program, with the aim of further developing and advancing it.

Understanding health disparities means to understand patients’ conditions and circumstances in different environment levels and to understand the, sometimes complex, interrelations between the environments levels. Extracting this complex and interrelated information required qualitative methods. Being a home visiting course, where learning is based on students’ experience of meeting patients at home and managing the home visit strengthen the need for qualitative methods. Since we had quantitative data relevant for the understanding of the course implementation and impact we employed a Concurrent Mixed Methods Design [27]. Quantitative data collected during the home visit by the student was supplemented by stakeholders’ views and the patients follow-up phone interview.

### Evaluation

#### Quantitative data

*Student characteristics* – were collected from a student survey at the start of medical school and included demographic data, knowledge of the Galilee residents’ customs and beliefs, and confidence handling a student-patient encounter around discharge.

*Patient characteristics* – students collected sociodemographic data, an evaluation of the complexity of the patients’ medical condition, health literacy, level of independence and support by completing two questionnaires, one during and one right after home visit. They submitted the questionnaires as part of their home visit report.

#### Quality and safety of the transition back home

1. The Medication Discrepancy Tool (MDT) [28] was completed by the students during home visit, aimed to identify discrepancies between patients’ medication regime and medications actually taken.
2. Patients’ perception of the adequacy of information received on discharge from hospital and their understanding of their medications was assessed at the home visit using the Hebrew version of the Care Transition Measure-3 (CTM3) [29, 30] and again in the follow-up interview. Reliability using cronbach’s alpha was 0.63 for home-visit questionnaire and 0.73 for follow-up interview. Since reliability was in the lower range of acceptability, we did not compute an overall score of the CTM3 but compared each item separately,

### Experience of the home visit

During the follow-up interview, the patients ranked students’ communication skills and their satisfaction with the service on a scale of 1–4.

### Qualitative data

#### Stakeholders (students, heads of department and patients) views

3. *Student interviews*– semi-structured interviews were conducted by one ETGAR faculty (DS) with 25 students, fifteen of them women students, representing all affiliated hospitals. Most interviews were during students’ second clinical year. At first ten students who were more involved in the course were purposely selected. The last fifteen were selected to balance and represent all affiliated hospitals. The interview guide questions related to the entire service, focusing on students’ experience, and learning while taking the course. For example: describe a “typical home visit”, what did you learn about the patient that you were not aware of in hospital, how did the course experience change your views of hospitalized patients (interview guide is attached as Appendix 1).. A code book was built according to the first 12 interviews, themes were updated after analyzing seven more interviews. Analysis of the last six interviews found no new themes and we assumed saturation. All of the students consented to be interviewed and recorded before starting the interview. The interviews, as all other interviews in this study, were done in Hebrew. Students’ interviews were about thirty minutes long.
4. *Student focus groups* – Eleven focus groups were conducted using single-category design [31]: five in 2017 and six in 2018, 37 and 45 students over all respectively. They were held as part of the last tutorial and the groups were students’ organic groups, their clinical rotation groups. The focus groups were held in two hospitals and the participants brought experience from all affiliated hospitals. Students were asked to reflect and share their experience of home visits and caring for patients and to offer the hospital management their recommendations for improving the discharge process. We expected that meeting the students in their organic group, during the course, where they can share and brainstorm will extract ideas and thoughts not always evident in one-on-one interview, and will contribute to the richness of this study. The focus groups were mediated, and documented according to an agreed and trained guide, by ETGAR faculty DS, MCJR and SS.
5. *Interviews with heads of department* – Fifteen heads of departments, who hosted most of ETGAR students groups were identified. Thirteen, representing three affiliated hospitals, agreed to be interviewed. An independent researcher conducted the interviews, led by an agreed interview guide.
6. *Patient interviews*–The interviewer summarized the main issues raised by the patients and any difficulty or barriers the interviewer had conducting the interview. Since we analyzed the interviews after completing all interviews and each interview contained only one or two topics, we analyzed all interviews to grasp a broad picture of patients’ view.

### Data analysis

We employed descriptive statistics in analyzing patients’ characteristics, medication discrepancies, and closed questions in the follow-up interviews. Change over time in patients’ CTM score was examined using Wilcoxon Signed Ranks for paired data.

Interviews with students and heads of department were recorded, transcribed verbatim, and were subject to content analysis directed by the grounded theory model. The student analysis was based on an explanatory framework derived from the social-ecology approach [18]. Heads of department analysis was based on an exploratory framework extracting their views on course impact and implementation. Focus groups and patients’ follow-up interviews were subjected to thematic analysis for deriving main themes addressed by students and patients. Frequency scale coding [27] was used for patients’ interviews to explore weight and intensity of patients’ views on the home visit impact.

To ensure trustworthiness, an independent interviewer conducted the interviews with heads of department, and two investigators analyzed it independently, reaching agreement between them. Independent interviewers conducted the patients’ interviews. Two ETGAR Faculty held and documented the focus groups each year, three Faculty overall (DS, SS, MR). One investigator (DS) conducted and analyzed the student interviews, and its results were triangulated with the other qualitative and quantitative findings.

## Results

### Quantitative results

#### Student characteristics

Two hundred and sixty-two of the 306 students (86%) completed the survey at the start of medical school. Mean age was 27.6 (3.5) years, 146 (55%) were female; 248 (94%) were Jewish and 211 (80%) born in Israel. Starting the course, students reported medium-low confidence in handling patient encounters 3.0 (0.9), and poor knowledge of customs and beliefs of Galilee residents 2.5 (1.0), (1–5 scale).

#### Patient characteristics

Four hundred eighty-fivepatients were visited with full documentation; fifty visits failed due to patients’ declining the visit following discharge. Patients’ sociodemographic characteristics are shown in Table 1. Of the 392 adult patients, 345 (88%) had at least one attribute of disadvantage, and 271 (69%) two or more. They had on average 4.6 (3.1) medical problems, were discharged on 5.5 (4.0) medications and were given 5.8 (2.6) post discharge instructions.

**Table 1:** Sociodemographic characteristics of patients visited by students in three academic years, 2016–2019

Patients’ characteristics	N (%)
*All patients (N = 485)*	
Reported income below average (*n* = 413)^a^	222 (54%)
Immigrants (*n* = 477) ^a^	155 (32%)
Non-Jewish patients (*n* = 465) ^a^	91 (20%)
*Adult patients’ only (excluding pediatric department) (N = 392)*	
< 12 years education (*N* = 361) ^a^	105 (29%)
Age 60+ (*N* = 384)	236 (61%)
Chronic medical condition (*N* = 386)	316 (82%)
Poor Hebrew proficiency (*N* = 384)^b^	51 (13%)
Inadequate support (available assistance at home) (*N* = 381) ^b^	78 (20%)
Low level of independence (*N* = 384) ^b^	144 (38%)
Poor knowledge of treatment (*N* = 387) ^b^	72 (19%)

^a^Patients’ report

^b^students’ post-visit evaluation

#### Quality and safety of the transition back home

Students identified medication discrepancies in 42% of home visits (148 of 357 adult patients), involving 2.2 (1.9) medications when discrepancies were found. Patients ranked the adequacy of information provided by the hospital relatively highly at the start of the home visit and felt this improved significantly following the student’s visit (3.2 (0.96) vs 3.8 (0.57), Z = -7.12, *p* < .001) as did their understanding of their prescribed medications (3.3 (0.92) to 3.8 (0.59), Z = -6.46, *p* < .001) both on a scale of 1–4.

#### Experience of the home visit

Out of 437 recruited patients’ details handed to the interviewer, 346 interviews took place. Missing interviews were due to failure to contact the patient (39 patients), too long elapse time between home visit and interview (35 patients), and lack of consent form (17 patients). Out of the 346 interviews, seven patients died before the interview took place and 38 patients did not cooperate or did not remember the home-visit. Three hundred one patient interviews had eligible qualitative data. Two hundred eighty-eight of them had full quantitative data (CTM) paired with patients’ responses during home visits.

Patients ranked students’ communication skills as very good, 3.97 (0.17), with 263 (93%) giving the maximum score of 4. They recommended ETGAR highly as a routine service 3.96 (0.18); 279 (97%) gave the maximum score.

219 (73%) of the 301 interviewed patients reported they benefited from the visit, that students increased their understanding of their medical condition and treatment and provided emotional support.

### Stakeholders’ views

Triangulating the qualitative analysis findings, four themes emerged from stakeholders’ views: Contribution to learning; experience-based learning; professional identity formation; course implementation.

#### Contribution to learning

Heads of department and students highlighted ETGAR’s contribution to understanding patients’ conditions in the community and understanding patients in a holistic way.

Through meeting patients in their home environment students were exposed to SDOH such as housing, the living environment, socioeconomic conditions, and social support, and they learned, first-hand, how SDOH influences patients’ health.

*Arriving in the neighborhoods I was looking around, saying to myself ‘Wow I do not know this world’. At home you learn about patients’ daily routine, self-care, social support, and the consequence of lack of support. It is all connected. (male student, 2019).*

ETGAR was perceived as complementing hospital-based learning, exposing students to accessibility to care, living conditions, poverty, loneliness etc. which are rarely raised in hospital. Through meeting patients at home, students gained an understanding of how limited their view on patients’ lives while in hospital was. They learned how patients care for themselves away from direct health professional control, and were exposed to hospital-community gaps, learning that some gaps are rooted in inadequate discharge processes.

*I identified significant gaps, not necessarily in proper medical care, but in self-management of care at home, how to set appointments and where to seek care. It gave me an additional point of view, especially on elderly patients.* (*male student 2019)*

Students described how meeting patients at home gave them a more empathic and less judgmental way of thinking.

We all tend to judge others. Meeting a patient with diabetes, hypertension, and obesity, you wonder, how come they are so irresponsible not caring for themselves? Learning of patients' circumstances, the way they live and provide for their families you will probably stop being judgmental. (male student, 2019)

Students reflected on understanding patients’ needs to be heard, acknowledging that listening, addressing patients’ concerns, and plain-language communication are integral aspects of patient care.

The course also shifted students from being learners to advocates, requiring them to learn all aspects of the patient’s condition when preparing home visits. As one Head of department noted:

To teach (the patients) the students must learn, to gain a profound knowledge of the patient’s medical condition, symptoms, tests, treatment, follow up and complications. It is a golden educational opportunity to engage students in learning. (Head of pediatric department)

#### Experience-based learning

The course took the students out of their hospital comfort zone giving them almost sole responsibility for the conduct of home visits, an experience some considered as “jumping into the water”. Gaining experience, students found their own ways for managing the encounter, overcoming challenges and inconveniences. Although students received their white coats when starting the clinical year, some felt ETGAR made them really wear them. Caring for patients on their own raised understandable anxiety in some; working in pairs helped to reduce concerns. Completing their first home visit and realizing the task was doable and beneficial for the patient helped further.

Visiting the first patient was stressful. How should I approach patients? How will I answer questions? I hardly understood the discharge letter myself. … along the way, like training a muscle, I improved my knowledge, confidence, communication skills and management of the doctor-patient encounter. (female student 2019)

Students played an active role in improving patients’ condition through confirming patients’ understanding and treatment, which is often incomplete when patients leave hospital [32]. Home visits allowed this to happen, with time and space for students to build trust and provide attentive care and guidance.

#### Professional identity formation

Through meeting patients in hospital and at home, and witnessing patients’ experience of hospital and community care, the students gained insights regarding how they wished to care for patients. As doctors of the future, they saw the need to confirm understanding and adherence, to inquire about the circumstances that brought patients to hospital and the physical and social environment to which patients are discharged.

Home visits gave me a new perspective. When meeting patients in hospital I must look beyond this temporary phase and enquire about their home: living conditions, who lives with them, and support they have or need when leaving hospital. (male student 2019)

The students frequently referred to their duty to see patients in a holistic way. Listening, understanding patients’ circumstances, and comparing this to the communication practiced in hospital, they realized the importance of looking beyond patients’ diseases and clinical measures.

It added new perspectives on patients, especially older patients. As a doctor, you cannot focus on the illness only … You must learn to see the patient in a holistic way, to consider other aspects. (male student 2019)

Patients’ views echoed students’ and physicians’ views, acknowledging that students provided good guidance, improving their understanding, and their well-being and health over time. Listening attentively and using plain language were skills especially appreciated. Explanations of medications and tests and providing plain-language discharge letters improved patients’ knowledge and empowered them by giving them tools to manage their health.

The students provided me with a detailed document which contained information I did not know. I took it with me to the family doctor and when I was hospitalized again. The student went over my medications explaining what they are for and why I should take them*.*

Patients referred to the importance of meeting a professional who cares and made them feel heard. In their own words, they described patient-centered encounters, where students educated them while addressing their emotions and needs. In doing so students improved patients’ adherence to treatment.

The student answered kindly and patiently to my questions. He explained about the medications and found one I was not taking as instructed. Since then, I am trying my best to take the medications correctly*.*

#### Course implementation

Recruiting patients and accessibility to patients’ homes were the main challenges students faced, particularly for patients living in remote areas with limited public transportation. Some students admitted that they considered patients’ addresses when recruiting them. Although hardly evident in the data, students reported a tendency to avoid patients with low Hebrew proficiency.

Although students knew how to access medical or social services advice, they felt at times that they lacked clinical support and close supervision. Department staff conceded they needed to be better informed about the course, acknowledging that their support was needed to promote uniform training and learning.

ETGAR was viewed by students as a time and resource consuming course. Adding it to students’ clinical year duties, generated some opposition, most evident in the first year of implementation. Most of the students acknowledged ETGAR’s importance educationally, but some claimed it should not be part of the clinical years due to workload and a lack of perceived contribution to final examination success.

No one doubted the importance of the course, but it felt misplaced. (Why?) It is difficult to explain, we are all involved in learning clinical year stuff, and here comes a course not teaching us exam related material, I know it sounds a bit immature. (male student, 2017)

## Discussion

Triangulating three sets of views we found ETGAR contributed to learning making students witness, first-hand, the interaction between patients’ circumstances and health. Patient-centered communication skills were promoted, the hospital-community gap appreciated, and holistic perceptions of patients adopted. Students developed a sense of the doctors they wished to be in the future.

### Contribution to learning

Social ecology provides a holistic framework emphasizing the relationship between factors at differing environmental levels and patients’ health [20, 33]. We used this health-promotion model to develop ETGAR, exposing students to four environment levels affecting patients’ health. As anticipated, the exposure allowed students to learn about the interplay between SDOH and patients’ health in a way that is not possible in hospital alone. Students appreciated the need to look beyond the biomedical perspective; some concluding that it is the physicians’ duty to enquire after personal and social conditions as part of standard care. In social ecology terms, they identified the need to seek factors affecting patients’ life and health at all environment levels.

Although students’ medical and professional learning advances during the clinical years, it is not always the case with communication skills and patient-centered skills which may even deteriorate [14]. Fragmented patient-doctor relationships, inadequate time to learn from and with patients, and unstructured learning are possible reasons [34]. Through preparing and conducting four home visits, students practice and appreciate the importance of communication skills such as listening and plain-language. Providing a structured learning framework requiring long term relationship with the patients and meaningful encounters at home, the course gave students the opportunity to learn with and from the patients and promoted patient-centered encounters. Students commented on the need to utilize these skills in the future, suggesting that this service learning may contribute to positive attitudes toward a patient-centered approach in the long term.

### Experience-based learning

Student-delivered services as a means for medical education are not new. Similar to student-run clinics [35], our students served patients in a real-life situation but differed in having sole responsibility at the point of contact. Meeting patients at home gave added value and exposed students to patients’ self-care, living conditions and family and community support systems. As patients regained autonomy once home, students could learn more deeply about patients’ beliefs, personal and social circumstances, and gain new perspectives, the patient’s, on hospital care.

Students were given the opportunity to be ‘actors in performance’, the highest level of involvement according to the experience-based learning approach [36, 37]. The value was confirmed by students and Heads of department who described how students integrated skills and knowledge in caring for patients. A further desired outcome in experience-based learning is affective learning [37]. The adoption of an empathic approach, overcoming concerns, gaining confidence, and developing a sense of themselves as future doctors indicate students achieved this outcome.

### Professional identity formation

The third year of medical school is a time when students’ humanistic approach and empathy may particularly erode. High demands and workload imposed by the medical school, fewer and shorter patient encounters, and a scientific medical approach have been given as possible explanations [38]. Our findings suggest that ETGAR, through its didactic framework which directs students to engage in deep doctor-patient encounters [38, 39], may offer an approach that encourages retention of empathy.

### Course implementation

Hospital-based programs can be non-uniform and less controlled than pre-clinical programs [8]. Students’ clinical tutors were not always knowledgeable enough and available for challenges ETGAR students faced, therefor students sometimes lacked direct and immediate support in hospital despite curriculum level and program managers’ support [37].

This research demonstrates the utilization of the social ecology model, mostly used for health promotion programs [20], as the basis for a health disparities educational program. The research findings also suggest that caring for patient in ETGAR-like educational scheme offers a mean to ameliorate empathy erosion and the deterioration of patient centered communication who are prevalent in the clinical years [14, 38].

Our study has certain limitations. There was no control group, so although students and heads of department, all experienced educators, acknowledged the benefit of home-visits for learning, we should not assume the same learning outcomes would not have been achieved without this course. Importantly the outcomes were affirmed by patients, (although for consent reasons, these patients had received visits by students on scholarship rather than during the course). It is conceivable that scholarship students may have differed in attitudes and involvement, although this did not emerge as a factor in focus groups. Lastly, our conclusions regarding the quality of students’ communication skills were largely based on self-report, rather than observational data. Reassuringly the findings were confirmed by patients and heads of departments who reported patient-centeredness and empathy towards their ETGAR patients.

## Conclusions

ETGAR responded to the challenge of designing an educational program that addresses the amelioration of health disparities [6]. Students were provided with the opportunity to meet and serve vulnerable patients in hospital and at home, within a didactic framework to promote learning. Integrating ETGAR in the clinical years combined community and communication learning into a traditionally bio-medical setting, promoting integration into practice, and opened new perspectives on patients and patient care. Results indicate that ETGAR, an educational program based on the social ecological model, impacted on students’ understanding of SDOH, health disparities and patients’ condition in the community, and promoted patient-centered communication skills. As such ETGAR was perceived to complement hospital-based education. This course can serve as a model for medical schools wishing to implement a long-term experience-based health disparities program into the medical school curriculum.

## Supplementary Information

**Additional file 1.**

## Data Availability

The datasets used and/or analyzed during the current study are available from the corresponding author on reasonable request.
