# Supplementary material for: A social ecological approach to promote learning health disparities in the clinical years: impact of a home-visiting educational program for medical students
Source: BMC Med Educ. 2022 Sep 30;22:698. doi: 10.1186/s12909-022-03755-3 (PMC9524119; doi:10.1186/s12909-022-03755-3)
Supplement: Supplementary file 1 — Additional file 1. [file 12909_2022_3755_MOESM1_ESM.docx]

**Appendix 1 - ETGAR medical students interview guide**

My name is Doron. I am part of ETGAR team and I am interested in ETGAR as an educational program. I am looking for the impact ETGAR has on you and on your peers as medical students and future doctors.

Before starting I’m asking for your consent to record our conversation. I would like to emphasize that there are no correct or incorrect answers. I prepared a list of questions for this interview, please feel free to answer or skip any of them. This interview is discrete, all identifying details will be removed from its final analysis. I am interested in your point of view regarding this project. Anything you say will improve our understanding of this project, and nothing you say will have any influence on your status or achievements as a student. Do you have any questions?

Before starting, if you will feel uncomfortable and wish to end the interview or take a break, please do not hesitate to ask for it.

1. What were your expectations before starting the course?
2. Please recall the major challenges you faced during the course.
3. Patients’ recruitment – describe your experience of recruiting patents, challenges, successful or unsuccessful recruitments, preparations you made, techniques to approach patients.
4. Experience from meeting patients at home – how did you open the visit? Describe a typical visit, a situation where you felt uncomfortable and how did you resolve it? Home visits or events you that left a significant memory.
   Did you learn something new, you did not know in hospital, about a patient? (To ask specific questions regarding medical, personal and social support issues if needed).
   Issues you discovered at home in addition to those the patient reported on.
5. How did you prepare yourself for the home visit?
6. What was the impact of home visits on the way you perceived patients and their illness?
7. Plain language writing – describe the easier or more difficult tasks you had while transforming the discharge letter into a plain language letter.
   Describe your considerations regarding what to omit or emphasize in the letter.
8. What did you learn from ETGAR?
   To what extent did the course contribute to you as a future doctor? What added value did it have to community and communication courses you took in the pre-clinical years?
   How did you contribute to your patient and in what ways did they contribute to you / to your learning?
9. At the start there was objection to the course as a required course. Can you explain the source for this objection? What should be done to overcome these objections?
